# Biallelic inactivation of *SDHA* results in comorbidity of pediatric recurrent neuroblastoma and gastric stromal tumor

**DOI:** 10.1016/j.gendis.2023.101196

**Published:** 2023-12-15

**Authors:** Shen Yang, Shixuan Zhang, Wen Zhao, Libing Fu, Li Zhang, Chan Hon Chui, Ruolan Guo, Yan Su, Dayan Sun, Huanmin Wang

**Affiliations:** a Department of Oncology Surgery, Beijing Children's Hospital, Capital Medical University, National Center for Children's Health, Beijing 100045, China; b State Key Laboratory of Genetic Engineering, Collaborative Innovation Center for Genetics and Development, School of Life Sciences and Human Phenome Institute, Fudan University, Shanghai 200438, China; c Hematology Oncology Center, Beijing Children's Hospital, Capital Medical University, National Center for Children's Health, Beijing 100045, China; d Department of Pathology, Beijing Children's Hospital, Capital Medical University, National Center for Children's Health, Beijing 100045, China; e Shanghai Institute of Precision Medicine, Shanghai Jiao Tong University School of Medicine, Shanghai 200011, China; f Surgery Centre for Children, Mount Elizabeth Medical Centre, 228510, Singapore; g Beijing Key Laboratory for Genetics of Birth Defects, Beijing Pediatric Research Institute, MOE Key Laboratory of Major Diseases in Children, Genetics and Birth Defects Control Center, Beijing Children's Hospital, Capital Medical University, National Center for Children's Health, Beijing 100045, China; h Department of Neonatal Surgery, Beijing Children's Hospital, Capital Medical University, National Center for Children's Health, Beijing 100045, China; i MOE Key Laboratory of Major Diseases in Children, Beijing Children's Hospital, Capital Medical University, National Center for Children's Health, Beijing 100045, China

Neuroblastoma (NB) is a common pediatric extracranial solid tumor that exhibits varied characteristics, clinical features, and prognosis.^1^ Totally 1%–2% of cases show familial history with genetic links like *ALK*, *PHOX2B* mutations, and 1p36 or 11q14-23 locus deletions. Gastrointestinal stromal tumors (GISTs) are rare mesenchymal neoplasms of the gastrointestinal tract. Approximately 85% of pediatric patients with GIST lack oncogenic mutations in *cKIT* or platelet-derived growth factor receptor alpha (*PDGFRA*), and a majority of these are characterized by molecular alterations in the succinate dehydrogenase (*SDH*) family of genes.^2^^,^^3^ Genetic changes, germline mutations, and variant-phenotype links in NB and GIST remain largely unexplored. Here, we reported a case of biallelic *SDHA* variant and copy number deletion causing pediatric recurrent NB with GIST to enhance the understanding of this rare clinical scenario.

The proband was an 8-year-old girl born to consanguineous parents of Chinese origin, and she was the first child of the couple (Fig. 1A). The second child was a 2-year-old boy without any obvious health issues. The parents and other known relatives did not have congenital malformations or tumors. The patient presented with abdominal pain and her abdominal enhanced computed tomography (CT) examination revealed a right adrenal gland mass with surrounding lymph node metastasis and gastric wall lesion (Fig. 1C (1)). Positron emission tomography (PET)/CT suggested hypermetabolic lesions located in the right adrenal gland and the surrounding lymph nodes, as well as the wall of the stomach (Fig. 1C (2)). Tumor marker examination showed that serum neuron-specific enolase was increased (180 ng/mL; normal range: <25 ng/mL), and the levels of urinary vanillylmandelic acid and homovanillic acid were normal. No tumor was found in the bone marrow. Core needle biopsy pathology of the right adrenal mass indicated poorly differentiated NB. No abnormalities were found in the 1p36, 11q23, and *MYCN* genes detected by fluorescence *in situ* hybridization. The stage of the International Neuroblastoma Risk Group Staging System (INRGSS) was L2, and the INRG group was intermediate-risk. After 2 courses of chemotherapy, retroperitoneal mass and stomach mass resection were performed. The postoperative pathology confirmed that NB (right adrenal gland) changed after chemotherapy (Fig. 1C (3)). In addition, the pathology of gastric mass was GIST, low-grade malignancy, with no *cKIT* or *PDGFRA* gene mutation detected in tumor tissue (Fig. 1C (4)). Furthermore, 3 courses of postoperative chemotherapy were performed. Subsequently, the patient received retroperitoneal external radiotherapy with a total of 25.2 Gy (1.8 Gy/time, 14 times) for NB, and all treatments were stopped at the age of 8.5 years old. The patient was followed up regularly until 12 years old, and repeated imaging examinations showed no recurrence (Fig. 1B). When the girl was 12 years and 2 months old, that is, nearly 4 years after stopping all treatments, abdominal ultrasonography and enhanced CT examination showed tumor lesion in the tail of pancreas, and 2 lesions in the stomach body and the stomach wall of the sinus (Fig. 1C (5)). Tumor markers were normal and no tumor invasion was found in the bone marrow. PET/CT indicated hypermetabolic masses in the tail of the pancreas and stomach wall (Fig. 1C (6)). Pathological diagnosis after tumor resection showed pancreatic tail NB (poorly differentiated) (Fig. 1C (7)), combined with stomach GIST (Fig. 1C (8)). Molecular diagnosis indicated no abnormality in 1p36, 11q23, *MYCN*, and *ALK* of NB recurrence lesion. No mutation of *cKIT* and *PDGFRA* genes was found in the GIST recurrence lesion. Considering that the patient had local recurrence of NB and no signs of distant metastasis and complete surgical resection, the intermediate-risk chemotherapy of NB, plus radiotherapy and retinoic acid-induced differentiation therapy, was given. At present, after more than 14 months of follow-up, the child is in complete remission.

**Figure 1:**
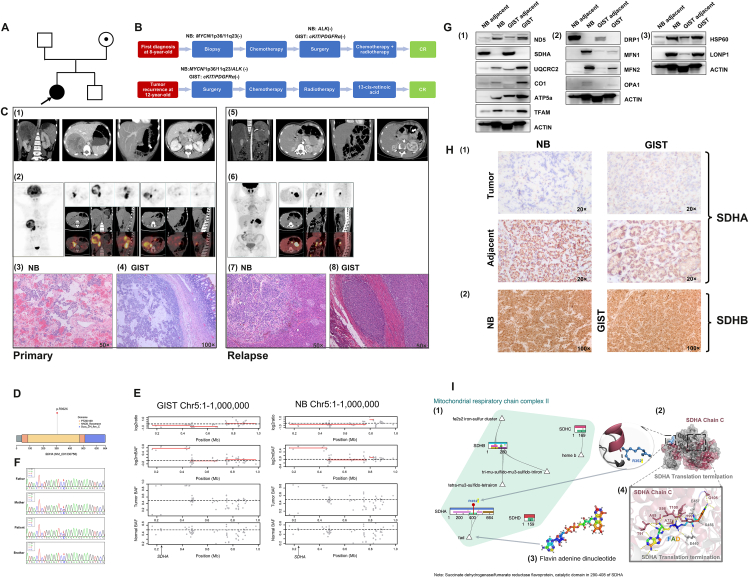
Biallelic inactivation of a germline truncating variant and a somatic copy number deletion in *SDHA* results in the comorbidity of pediatric recurrent neuroblastoma (NB) and gastrointestinal stromal tumor (GIST). **(A)** Pedigree chart of the proband's family. Males are depicted as squares and females as circles. Black-filled symbols represent individuals who are clinically affected. A dot in squares or circles indicates the carrier. The proband who underwent whole exome sequencing is indicated by an arrow. **(B)** The flow chart of the patient's clinical course of treatment. CR, complete response. **(C) (1)** Abdominal enhanced computed tomography (CT) at the time of initial diagnosis. **(2)** Whole-body positron emission tomography (PET)/CT at the time of initial diagnosis. **(3)** A postoperative pathological investigation of the retroperitoneal mass confirmed that NB (right adrenal gland) changed after chemotherapy. **(4)** The pathology of gastric mass was low-grade malignancy GIST. The arrows indicate a GIST lesion. **(5)** Abdominal enhanced CT at the time of tumor recurrence. **(6)** Whole-body PET/CT at the time of tumor recurrence. **(7)** The pathology of the pancreatic mass was pancreatic tail NB (poorly differentiated). **(8)** The pathology of gastric mass was GIST. The arrows indicate the recurrent GIST lesion. **(D)** The amino acid changes of the germline truncating variant on SDHA protein domains derived from the PFAM database. **(E)** The chromosomal region of the somatic loss-of-heterozygosity identified by saasCNV. The copy number variation regions were jointly segmented by read depth (log2ratio) and allele-specific read depth (log2mBAF). **(F)** Sanger sequencing chromatogram depicts the germline mutation of *SDHA*. **(G) (1)** The protein expression levels of mitochondrial complex subunits and the transcription factor of mitochondrial complex subunits of the adjacent tumor tissue and tumor tissue of recurrent GIST and NB. **(2)** The expression of mitochondrial fusion- and mitochondrial fission-related proteins. **(3)** The expression of mitochondrial quality control-related proteins. **(H) (1)** Immunohistochemistry (IHC) results of the SDHA expression in recurrent NB and GIST and adjacent tissues. **(2)** IHC results of the SDHB expression in recurrent NB and GIST. **(I) (1)** Mitochondrial respiratory chain complex II with acting substrates and acting structural regions. **(2)** The rs746165168 (Arg^352^Ter) mutation in SDHA and the structural region of the protein, where the grey part represents translation arrest and the purple part represents the normally translated peptide chain. **(3)** Flavin adenine dinucleotide (FAD) molecular structure. **(4)** Interaction of the SDHA protein C-chain with FAD, with yellow for hydrogen-kin, grey for translation-terminated amino acids, and purple for normal translation amino acids.

After tumor recurrence, whole exome sequencing was used with the patient's genomic DNA from PBMC and relapsed tumors to decipher disease-causing variants. The variant analysis found *SDHA* (NM_001330758, p. R352X) germline truncating variant, novel in Clinvar and HGMD (Fig. 1D). The pedigree analysis showed the variant was maternally inherited, with the mother being heterozygous (Fig. 1E). The mother carries a truncating variant in SDHA without illness. Thus, the proband tumor may result from a somatic *SDHA* truncating variant and other mutations. However, there were no somatic mutations in *SDHA* found in the tumor tissues. Furthermore, the analysis of somatic copy number alterations revealed that a loss-of-heterozygosity (LOH) region at chr5:163205-457955 was found in both of the two tumor tissues (Fig. 1F). Coincidentally, the LOH region covered the whole gene of *SDHA* and located within the other homologous chromosome compared with the *SDHA* truncating variant. NB and GIST link to the *SDHA* germline variant and LOH, aligning with the two-hit hypothesis.

SDHA, vital for mitochondrial complex II, was detected in GIST, NB tumors, and adjacent tissues. To unravel the mitochondrial role in concurrent GIST and NB, we assessed SDHA expression in recurrent tumor tissues and adjacent ones, revealing stark SDHA loss in tumors alongside up-regulated subunits of mitochondrial complex I (ND5), complex III (UQCRC2), complex IV (CO1), and complex V (ATP5a) (Fig. 1G (1)). Immunohistochemistry (IHC) confirmed SDHA absence in tumors, contrasting with adjacent tissues (Fig. 1H (1)). Notably, mitochondrial fusion proteins (MFN1, MFN2, OPA1) increased, fission protein (DRP1) decreased (Fig. 1G (2)), while mitochondrial quality control-related HSP60 and LONP1 were elevated (Fig. 1G (3)). Unlike SDHB expression loss associated with epigenetic mechanisms,^2^ our study did not find reduced SDHB expression via IHC (Fig. 1H (2)). Our findings collectively suggest diminished SDHA and skewed mitochondrial biogenesis in GIST and NB.

Aberrant mitochondrial biogenesis contributes to tumorigenesis. SDHA, part of mitochondrial complex II (Fig. 1I (1, 2)), transfers electrons, impacting the citric acid cycle. Reduced complex function causes succinate buildup, inhibiting dioxygenase and promoting HIF1 catabolism. This up-regulates genes (*e.g.*, IGF-1, VEGF) promoting tumor formation. SDH deletion alters amino acid metabolism and mitochondrial dysfunction, elevating reactive oxygen species levels.^3^^,^^4^

Previously overlooked rs746165168 (C > A) *SDHA* nonsense mutation causes early termination at *SDHA* segment C (353–664), impairing function. Our study links rs746165168 to NB and GIST. We found that functionally incomplete SDHA (Arg^352^Ter) could impact the flavin adenine dinucleotide catalytic region (Fig. 1I (3)), affecting redox reactions via hydrogen bonding (E^457^, E^440^, S^456^; Fig. 1I (4)).

Previous research links *SDHA* germline variants to pheochromocytoma, renal tumor, and GIST tumor. Gault et al reported *SDHA* mutations in NB, breast, colon, kidney, melanoma, uterine, prostate, endometria, bladder, and GIST. *SDHA* mutations show low penetrance^5^ (<0.3% in healthy population). Our study confirms SDHA's role in GIST and NB via IHC staining, second hits, LOH, and altered mitochondrial protein expression, supporting heightened biogenesis for energy.

Mitochondrial fusion and fission impact tumorigenesis. Philley et al linked mtDNA depletion in prostate cancer to increased MFN1 and MFN2 expression. OPA1 overexpression, found by Liu et al, sustained cancer stemness in non-small cell lung cancer. Our study revealed heightened mitochondrial fusion in NB and GIST, implying better function. Mitochondrial unfolded protein response occurs during protein misfolding. In pancreatic cancer, Liu et al linked LONP1 silencing to suppressed progression. HSP60 up-regulation by Zhou et al enhanced oxidative phosphorylation and Erk1/2 activation, while its inhibition curbed prostate cancer. Targeting unfolded protein response could impede NB and GIST development.

Somatic mutations show a close resemblance between NB and GIST, hinting at potential causal links. GIST and NB co-occurrence in children was reported. NB's association with GANT, a GIST subtype, is proposed. While molecular evidence is lacking, our LOH similarity findings could offer theoretical support.

This study presents a rare case, preliminarily exploring pathogenesis. Further research is needed to understand how *SDHA* loss promotes NB and GIST co-occurrence.

## Ethics declaration

The study was approved by the Ethics Committee of Beijing Children's Hospital, Capital Medical University, and performed according to the Declaration of Helsinki. The written informed consent for publication was obtained from the parents of the patient. Clinical information and samples were collected from the patient, the parents, and the brother, all of whom had given their prior informed consent to participate in the study.

## Author contributions

SY, HW, DS, and SZ designed most of the studies. SY, SZ, and WZ carried out much of the work together with LF, LZ, CC, and RG. SZ, LZ, and DS analyzed the data. SY, SZ, WZ, DS, and LZ wrote up the manuscript. All authors red and approved the publication of the final manuscript.

## Conflict of interests

The authors declare that they have no conflict of interests.

## Funding

This work was supported by the 10.13039/501100001809National Natural Science Foundation of China (No. 82293660, 82293665) and the Consulting and Research Project of the Chinese Academy of Engineering (No. 2019-XY-34).

## Data availability

The datasets generated during and/or analyzed during the current study are not publicly available due to personal privacy and genetic resources protection but are available from the corresponding author upon reasonable request.
