# Supplementary material for: Biallelic inactivation of SDHA results in comorbidity of pediatric recurrent neuroblastoma and gastric stromal tumor
Source: Genes Dis. 2023 Dec 15;11(6):101196. doi: 10.1016/j.gendis.2023.101196 (PMC11512112; doi:10.1016/j.gendis.2023.101196)
Supplement: Multimedia component 1 [file mmc1.docx]

**Materials and methods**

**Human sample collection**

This study was conducted according to Declaration of Helsinki principles and approved by the Medical Ethics Committee of Beijing Children’s Hospital. Clinical information (including the demographic information, laboratory test and imaging examination results, clinical course), and blood or tissue samples were obtained from the patient, parents, and brother. Informed consent has been signed by all the participants.

**Histology and immunohistochemistry**

Tissues were fixed in 10% buffered formalin for at least 24 hours, embedded into paraffin. Tissues were then sectioned into 5 μm slice and stained with hematoxylin and eosin (H&E). For immunohistochemistry, recurrent tumor and adjacent tissues were sectioned, deparaffinized, rehydrated, treated by epitope retrieval using EDTA (pH 9.0), and incubated at room temperature with 0.3% H_2_O_2_, before being blocked of non-specific immunoreactivity with goat serum. Furthermore, sections were incubated with primary antibody of anti-SDHA (1:800, ab14715, Abcam, UK) and anti-SDHB (OriGene, China) at 4 ℃ overnight. After rinsing three times with PBST, the sections were incubated with biotinylated goat anti-mouse secondary antibody (Vector Laboratories) and subsequently with streptavidin-labeled HRP (Vector Laboratories). The sections were finally visualized with 3, 3’-diaminobenzidine (DAB) substrate and counterstained with hematoxylin. Digital images were recorded by an Olympus BX53 microscope.

**Whole exome sequencing (WES) analysis**

In this study, the tumor tissues used for WES detection were relapsed GIST and NB. The original fastq files, fastqc and multiqc were used to check the quality, exclude low quality reads and remove connectors. Raw data with paired samples (tumor and blood) were aligned to the human genome (hg19) using BWA aligner v0.7.12. PCR duplicates were removed and sequence metrics were collected using Picard v1.13. Single-nucleotide variants (SNVs) and small insertions and deletions (Indels) were called by VarScan v2.4.4. The somatic mutations for the two tumor samples were identified by VarScan somatic mode with default options using the blood sample as the control, respectively. The variants identified in all the three samples were considered as germline variants. The variants were annotated by ANNOVAR, and those with minor allele frequency (MAF) < 0.1% in healthy populations including 1000 Genome, ExAC, and GnomAD and predicted as deleterious by one of the tools including SIFT, PolyPhen2, and MutationTaster were considered as potentially pathogenic. The somatic copy number alterations (sCNAs) were called by saasCNV, which jointly segmented the chromosomal regions based on log2 copy number ratio between tumor and normal samples (log2ratio) and log2 B-allele frequency (log2BAF) of the variant.

**SDS-PAGE, immunoblotting, and antibodies**

In this study, relapsed GIST and NB and adjacent tumor tissues were used for protein detection. Proteins were extracted with RIPA lysis buffer (CST, USA) supplemented with a protease-inhibitor cocktail (Sigma, USA). Proteins were separated by SDS-PAGE, and were blotted with antibodies listed as follows: anti-VDAC (1:1000, ab154856, Abcam, UK), anti-ND5 (1:1000, 55410-1-AP, Proteintech, China), anti-SDHA (1:1000, ab14715, Abcam, UK), anti-UQCRC2 (1:1000, ab14745, Abcam, UK), anti-CO1 (1:1000, ab14705, Abcam, UK), anti-ATP5a (1:1000, ab14748, Abcam, UK), anti-TFAM (1:1000, ab176558, Abcam, UK), anti-DRP1 (1:1000, 12957-1-AP, Proteintech, China), anti-MFN1 (1:1000, 13798-1-AP, Proteintech, China), anti-MFN2 (1:1000, 12186-1-AP, Proteintech, China), anti-OPA1 (1:1000, 27733-1-AP, Proteintech, China), anti-HSP60 (1:1000, 15282-1-AP, Proteintech, China), anti-LONP1 (1:1000, 15440-1-AP, Proteintech, China). Beta Actin (1:1000, 60008-1-Ig, Proteintech, China) antibody was used as an internal antibody.

**The impact of mutation sites on the protein structure**

All the structures were downloaded from the PDB [6VAX(*SDHA*)] for analysis. The continuous interface residues where R352 is located were defined as the active binding threshold with Flavin adenine dinucleotide for *SDHA* (P31040). Site-directed mutagenesis was also conducted in PyMOL. The termination mutation at R352 prevents protein translation after amino acid 352. In the structure of 6VAX, the binding region of Chain C with FDA marks potential hydrogen bond changes after protein translation termination.
